# Impact of Deficiencies in Branched-Chain Fatty Acids and Staphyloxanthin in* Staphylococcus aureus*

**DOI:** 10.1155/2019/2603435

**Published:** 2019-01-22

**Authors:** Hannah Braungardt, Vineet K. Singh

**Affiliations:** Department of Microbiology and Immunology, Kirksville College of Osteopathic Medicine, A.T. Still University of Health Sciences, Kirksville, MO 63501, USA

## Abstract

*Staphylococcus aureus *is a well-known human pathogen with the ability to cause mild superficial skin infections to serious deep-tissue infections, such as osteomyelitis, pneumonia, and infective endocarditis. A key to* S. aureus *infections and its pathogenicity is its ability to survive in adverse environments, especially at lower temperatures, by regulation of its cell membrane. Branched-chain fatty acids (BCFAs) and staphyloxanthin have been shown to regulate membrane fluidity and staphylococcal virulence. This study was conducted with the hypothesis that the simultaneous lack of BCFAs and staphyloxanthin will have a far greater implication on environmental survival and virulence of* S. aureus*. Lack of a functional branched-chain *α*-keto acid dehydrogenase (BKD) enzyme because of a mutation in the* lpdA* gene led to a decrease in the production of BCFAs, membrane fluidity, slower growth, and poor* in vivo* survival of* S. aureus*. A mutation in the* crtM *gene eliminated the production of staphyloxanthin but it did not affect membrane BCFA levels, fluidity, growth, or* in vivo* survival. A* crtM*:*lpdA *double mutant showed much slower growth and attenuation compared to individual mutants. The results of this study suggest that simultaneous targeting of the BCFA and staphyloxanthin biosynthetic pathways can be a strategy to control* S. aureus *infections.

## 1. Introduction

*S. aureus *is a common cause of skin infections and is also responsible for a myriad of complicated deep-tissue diseases such as pneumonia, endocarditis, osteomyelitis, and septic shock [1–3]. Treatment of* S. aureus *infections can be difficult due to its antibiotic resistance. Infections caused by methicillin-resistant* S. aureus *(MRSA) and vancomycin resistant* S. aureus *(VRSA) have limited treatment options [4–6]. As an alternative strategy, metabolic pathways critical for survival and virulence can be targeted as ways to reduce* S. aureus *infections and their severity [6].

In order to survive in harsh environments, bacterial pathogens may alter the fatty acid composition of their membrane to maintain homeostasis [7–9]. Fatty acids in* S. aureus *cell membranes include saturated- and unsaturated-straight chain fatty acids (SCFAs) and branched-chain fatty acids (BCFAs) [10–12]. Levels of these fatty acids, especially BCFAs, change in response to fluctuations in growth environments such as temperature, humidity, and nutrient availability [10]. The enzyme, branched-chain *α*-keto acid dehydrogenase (BKD), catalyzes the committed step in the metabolic pathway that yields BCFAs. Lack of BKD enzymatic activity decreases BCFAs in* S. aureus *cell membrane which hinders the growth of this bacterium at low temperatures [11, 12].

An additional key regulator of* S. aureus *virulence and membrane fluidity is the carotenoid pigment, staphyloxanthin [13, 14]. Its production is mediated by enzymes encoded by five genes (*crtOPQMN*) present in a cluster in the staphylococcal chromosome [13, 14]. Inactivation of the genes in this cluster abolishes staphyloxanthin production which results in colorless colonies of this bacterium [13].

While a higher level of BCFAs leads to an increase in fluidity, an increase in carotenoid production decreases the membrane fluidity [15–18].* S. aureus *cells deficient in staphyloxanthin are more susceptible to the killing by neutrophils and are more easily cleared from infected tissues than* S. aureus *with staphyloxanthin [13]. It has been postulated that staphyloxanthin plays an important role in* S. aureus* virulence by conferring protection of the bacterial cells from oxidants generated during respiratory burst inside the phagocytic cells [13]. Recently, the staphyloxanthin pathway has been studied for its potential as a drug target to control* S. aureus* infections [19].

We recently noted an increased production of staphyloxanthin in BKD-deficient* S. aureus* cells with decreased level of BCFAs [12]. It was speculated that this increase in staphyloxanthin production compensated for a more rigid membrane due to a decreased membrane BCFAs [12]. The findings of this study highlight the roles of BCFAs and staphyloxanthin and their significance as antimicrobial targets to control infections caused by* S. aureus*.

## 2. Materials and Methods

### 2.1. Ethics Statement

Animal studies were conducted as per a protocol approved by the Institutional Animal Care and Use Committee of A.T. Still University-Kirksville College of Osteopathic Medicine.

### 2.2. Bacterial Strains, Antibiotics, and Growth Conditions

The bacterial strains used in this study are shown in Table 1. All cultures were grown aerobically at 37°C in tryptic soy broth (TSB) or on tryptic soy agar (TSA). Antibiotics, erythromycin (10 *μ*g ml^−1^) and kanamycin (100 *μ*g ml^−1^), were used when needed. Additionally, liquid bacterial cultures were grown in a shaking incubator at 220 rpm, and plated cultures were incubated for 24-48 h.

### 2.3. S. aureus crtM and lpdA Mutants

The* lpdA *mutant used in this study has previously been described [11]. A* crtM *mutant of* S. aureus* strain JE2 (NE1444) was obtained from the Nebraska Transposon Mutant Library. Mutation in* crtM* gene was subsequently transferred into* S. aureus* strain SH1000 using a phage transduction procedure and verified by PCR. To create* crtM*:*lpdA* double mutant, the* crtM* mutation was transduced into the* lpdA* mutant of* S. aureus* strain SH1000.

### 2.4. Complementation of* lpdA *and* crtM* Mutant

Complementation of the* lpdA *mutant was accomplished as previously described [11]. For complementation of the* crtM* mutant, a 6310 bp DNA fragment was PCR amplified using primer pairs (5′-GGTACCATCGCCATTCACCGTTATGT-3′ and 5′-TCTAGATGTTGGTGGGAAAATTGGTT-3′) and* S. aureus* SH1000 genomic DNA as template. This amplicon represents a fragment starting 436 nt upstream of the* crtO* gene, the entire carotenoid pathway encoding genes (*crtOPQMN*), and 337 nt downstream of the* crtN* termination codon. The amplicon was cloned into the shuttle plasmid pCU1 [21] which was then placed into the* crtM *mutant strains of* S. aureus *strain SH1000. Complementation was verified by dark pigmentation of the* crtM *mutants.

### 2.5. Growth Kinetics of the* crtM*,* lpdA*, and* crtM*:*lpdA *Mutants and Wild-Type Strain SH1000

Overnight cultures of these strains were diluted 1:10 and the absorbance of the diluted culture was measured spectrophotometrically. These overnight cultures were then diluted in a 250 ml Erlenmeyer flasks containing 50 ml of fresh TSB with loose metal caps for proper aeration. Dilutions were made 1:100 if the overnight cultures showed an OD_600_ of 6.0 or adjusted accordingly. The culture flasks were placed in a shaking incubator. Optical density (OD_600_) of each culture was determined spectrophotometrically every 30 minutes and continued until cultures reached the stationary phase. The generation time of growth of different* S. aureus* strains was calculated as previously described [22]. The growth rate (*μ*) was calculated using the formula (ln OD_2_ - ln OD_1_)/(t_2_ - t_1_) in which two time point ODs from 120 (t_1_) and 210 min (t_2_) were used, and the generation time was then calculated using the formula (ln 2/*μ*) as described previously [22].

### 2.6. Analysis of the Membrane Fatty Acid Composition in* crtM*,* lpdA*, and* crtM*:*lpdA* Mutants and the Parent SH1000

The membrane fatty acid composition was determined as previously described [11, 12]. Bacterial cells grown in TSB were harvested during mid-exponential growth phase (OD_600_ = 0.6). After washing three times with cold distilled water, cell pellets were frozen and shipped under dry ice for the analysis of membrane fatty acids to Midi Microbial Identification system (Newark, DE).

### 2.7. Carotenoid Levels in* crtM*:*lpdA *Double Mutant and the Complemented Strains

Quantification of the carotenoid pigment was carried out using a methanol extraction procedure as previously described [10]. Overnight cultures were diluted (1:100) and added to a flask containing 50.0 ml fresh TSB. The cultures were allowed to grow shaking at 37°C for 48 h. Cells from 5.0 ml culture were pelleted by centrifugation and washed twice with cold water, and the wet mass of the bacterial cell pellet was measured. Carotenoid pigments from these cells were then extracted after resuspension in 3.0 ml of methanol and incubation at 55°C for 3 min with intermittent agitation. A_465_ of the cell-free supernatant was then measured as level of carotenoid production, which was then normalized against the wet bacterial cell mass.

### 2.8. Determination of Membrane Fluidity of the* crtM*,* lpdA*, and* crtM*:*lpdA *Mutants and Wild-Type Strain SH1000

Membrane fluidity was measured as recently described [12]. Overnight cultures were diluted 1:100 and were grown at 37°C with shaking until an OD_600_ of 1.0 was reached. The cultures were then centrifuged to collect the bacterial cells that were washed twice with phosphate buffered saline (PBS) solution before being resuspended in PBS to an OD_600_ of 1.0. To this suspension, 4 *μ*l of 1,6-diphenyl-1,3,5-hexatriene (DPH) was added and the fluorescence polarization was measured using a spectrofluorometer (Fluorescence Spectrometer LS-55; Llantrisant, UK).

### 2.9. Survival of* lpdA*,* crtM*, and* crtM*:*lpdA *Mutants in Comparison to Wild-Type SH1000 in Mice

*In vivo* survival experiments were carried out as recently described [23]. Cultures of these strains were grown individually to mid-log phase (OD_600_ = 0.6) in TSB. Bacterial cells were then harvested by centrifugation, washed three times with 1% TSB, and resuspended in 1% TSB. Cell viability in the suspension was determined by serial dilution and plating. Three parallel mixtures of wild-type with* lpdA*,* crtM*, or* crtM*:*lpdA* mutants were prepared (1X10^7^ CFU ml^−1^; ~60/40 percent mixture of mutant/wild-type) in 1% TSB, and 0.2 ml of this suspension was injected into the peritoneal cavity of Swiss white Hla (ICR) CVF female mice (16-20 gram) (Hilltop Lab Animals, Inc.) with a 26-gauge needle fitted to a 1 ml syringe. At 8 and 24 h, the mice were euthanized and the liver and spleen were aseptically removed. Excised livers and spleens were homogenized in 2 and 1 ml of 1% TSB, respectively, using a glass tissue grinder fitted with a glass pestle. The tissue homogenates were serially diluted, plated on TSA and TSA containing appropriate antibiotics, and allowed to grow overnight by incubation at 37°C. The bacterial colonies growing in the presence of antibiotics were compared to the total number of colonies on TSA plates to calculate the fraction of mutants in the bacterial population in the infected tissues. The ratio of mutants in the infected tissues was then compared to the ratio of mutants in the inoculum used to infect the mice as previously described [23].

### 2.10. Statistical Analysis

Significance is determined using a one-way ANOVA and with a* p* value ≤ 0.05. For wild-type versus mutant in the mouse survival experiment, data were evaluated using one-sided proportion ratio with a Bonferroni correction using R studio.

## 3. Results

### 3.1. Growth Kinetics of the Mutant and Wild-Type Strains SH1000

**Figure 1:**
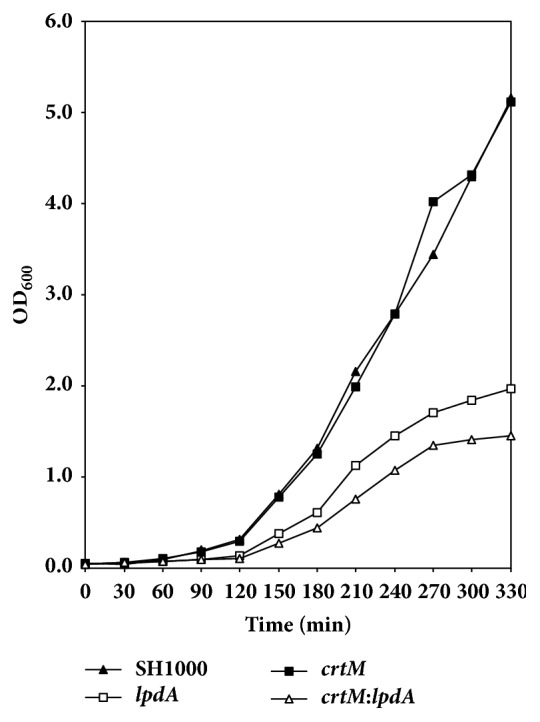
**Growth analysis of* S. aureus *strain SH1000 and derivative* lpdA* and* crtM* mutants. **Growth was measured every 30 min (OD_600_) until stationary phase was reached. Values represent an average of two independent experiments.

**Figure 2:**
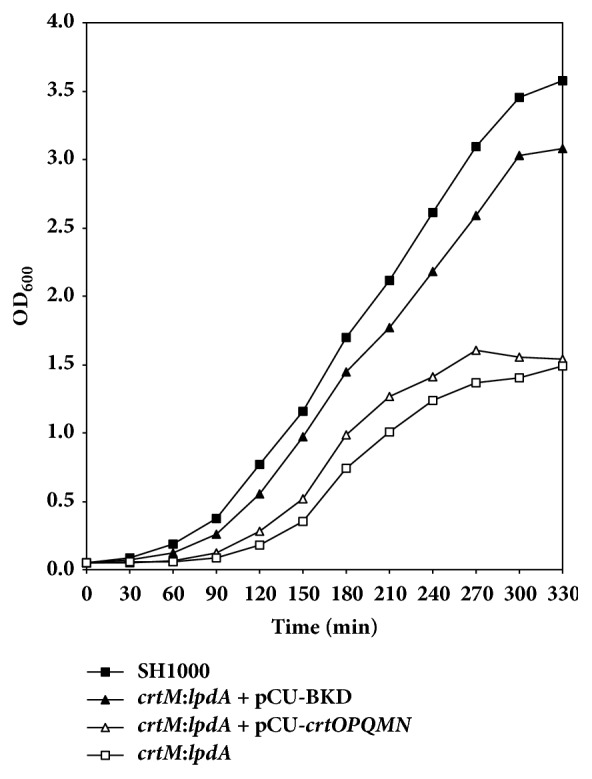
**Growth analysis of* crtM*:*lpdA* double mutant and the mutant complemented with BKD or* crt* gene cluster* in trans*. **Growth was measured every 30 min (OD_600_) until stationary phase was reached. Values represent an average of two independent experiments.

A comparison of the growth kinetics of the wild-type SH1000 and the* crtM* and* lpdA* mutants showed a decreased growth rate of the* lpdA *mutant (Figure 1, Table 2). On the other hand, the* crtM *mutant of the* S. aureus* strain SH1000 showed no change in growth in comparison to the wild-type (Figure 1, Table 2). The growth of the* crtM*:*lpdA* double mutant was significantly slower than the wild-type* S. aureus *as well as the* lpdA* individual mutant at every time point (Figure 1, Table 2). When the* crtM*:*lpdA *was complemented with a plasmid containing the entire* crt* operon, the growth of the complemented strain remained slower like the growth of the* lpdA* mutant (Figure 2). However, when the* crtM*:*lpdA *mutant was complemented with the entire* bkd *gene locus on plasmid pCU1, the growth of the complemented strain was restored to the level of the growth rate of the wild-type* S. aureus* strain SH1000 (Figure 2).

### 3.2. Analysis of the Membrane Fatty Acid Composition of Wild-Type* S. aureus* Strain SH1000 and Its Derivative* crtM*,* lpdA*, and* crtM*:*lpdA *Mutants

Cell membrane fatty acid compositions of* S. aureus *wild-type SH1000 and its isogenic mutants are shown in Table 3. In wild-type* S. aureus*, the BCFAs accounts for 70.7% of the total membrane lipids. Fatty acid profile of the* crtM *mutant of* S. aureus* SH1000 is similar to the fatty acid profile of the parent strain with about 70% BCFAs (Table 3). The* lpdA *mutant, on the other hand, showed a significant deficiency in BCFAs and represented only 35.1% of the membrane lipids (Table 3). Fatty acid profile of the* crtM*:*lpdA *double mutant was similar to the* lpdA* individual mutant of* S. aureus* SH1000 with a significantly lower BCFA level (42%) compared to their levels (70.7%) in the wild-type* S. aureus* (Table 3).

### 3.3. Carotenoid Levels in* crtM*:*lpdA *Double Mutant and the Complemented Strains

**Figure 3:**
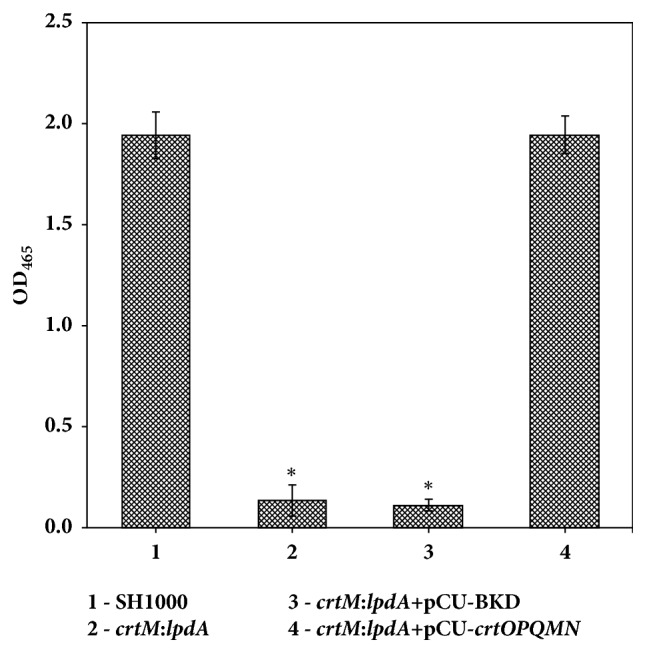
**Staphyloxanthin levels in* S. aureus *strain SH1000 and isogenic mutants. **Carotenoid pigments were methanol extracted from 48 h grown cultures and estimated spectrophotometrically by quantifying OD_465_. Values represent average of three independent experiments ± standard deviation (*∗* significant at* p*≤.05).

Inactivation of the* crtM *gene resulted in a nonpigmented* S. aureus* cell. In these experiments both the* crtM *and* crtM*:*lpdA *mutants were white and showed minimal carotenoid production (Figure 3). The wild-type* S. aureus* SH1000 and the* crtM*:*lpdA* double mutant complemented with* crt* gene locus showed restored levels of staphyloxanthin (Figure 3). Conversely, when the* crtM*:*lpdA* double mutant was complemented with the BKD gene cluster, there was no restoration in the mutant in terms of its ability to produce staphyloxanthin (Figure 3).

### 3.4. Membrane Fluidity of the* crtM*,* lpdA*, and* crtM*:*lpdA *Mutants and the Wild-Type* S. aureus* Strain SH1000

**Figure 4:**
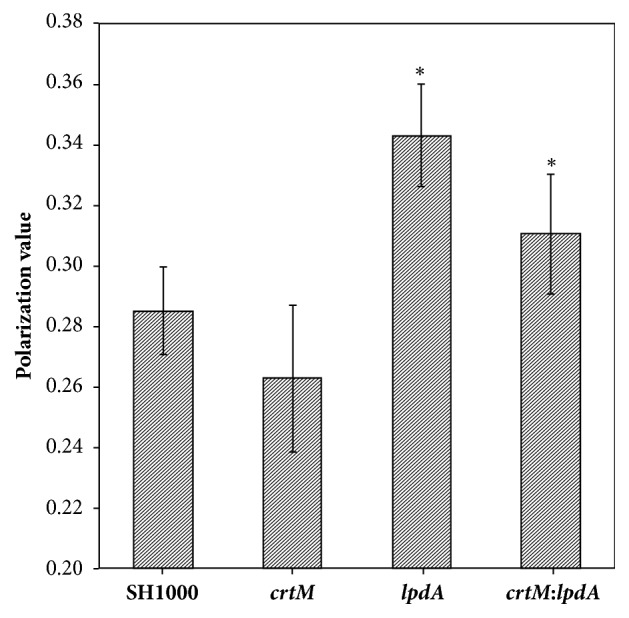
**Membrane fluidity of* S. aureus* strain SH1000 and derivative* lpdA*,* crtM*, and* crtM*:*lpdA* mutants.** Cells from mid-exponential phase cultures were washed and incubated with DPH. Membrane fluidity was measured with a spectrofluorometer. Values represent average of three independent experiments ± standard deviation (*∗* significant at* p*≤.05).

A mutation in the* lpdA* gene resulted in significant decrease in the membrane fluidity of the mutant bacteria (Figure 4). Polarization value recorded for the* lpdA* mutant was significantly greater when compared to the polarization value for the wild-type* S. aureus* suggesting a more rigid membrane. In contrast, the* crtM *mutant showed polarization value slightly lower when compared with the polarization value for the wild-type* S. aureus* strain SH1000 cells, but this change was not statistically significant. The* crtM*:*lpdA *double mutant was slightly more fluid than the* lpdA* mutant, but still significantly less fluid than the wild-type* S. aureus* strain SH1000.

### 3.5. Survival of* lpdA*,* crtM*, and* crtM*:*lpdA *Mutants in Mice

**Figure 5:**
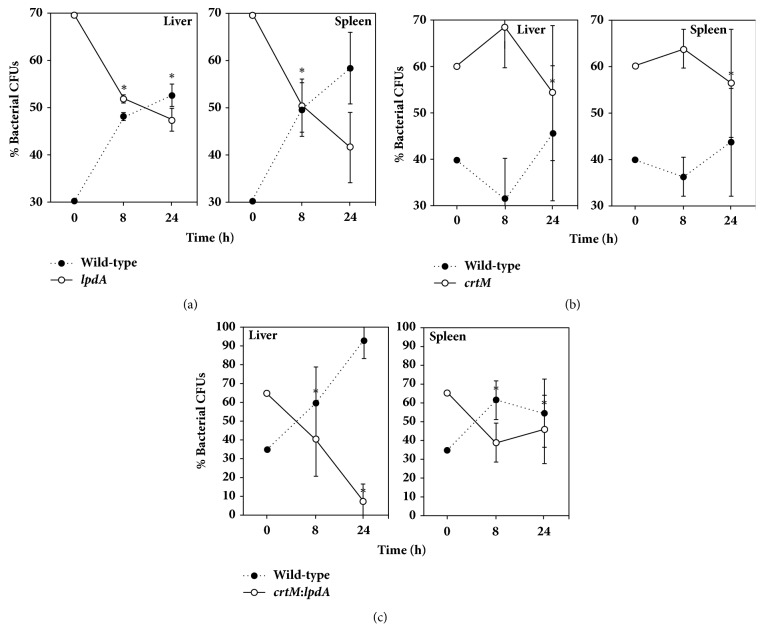
**Survival in mice. **Approximately 2X10^6^ bacteria were injected (~60:40 ratio of mutant to wild-type). Mice were sacrificed after 8 and 24 h. Values represent average of three independent mouse tissues ± standard deviation (*∗* significant at* p*≤.05). CFUs – colony forming units. (a)* lpdA* mutant:wild-type SH1000; (b)* crtM* mutant:wild-type SH1000; (c)* crtM*:*lpdA* mutant:wild-type SH1000.

In these bacterial survival studies in mice, a mixture of wild-type SH1000 with either* lpdA*,* crtM*, or* crtM*:*lpdA *mutants was injected into the peritoneal cavity. These mixtures were biased (~60% mutant and ~40% wild-type) to better assess any decrease in survival due to mutations in* crtM* or* lpdA* genes. In mice infected with the mixture of the wild-type and the* lpdA* mutant, the fraction of the mutant decreased with time in both liver and the spleen tissues compared to its fraction in the mixture used to infect the mice (Figure 5(a)). In contrast, there was no change in the survival of the* crtM* mutants as its ratio in the bacteria recovered from both liver and spleen tissues of the infected mice was similar to the ratio of* crtM* mutants in the injected mixture (Figure 5(b)). The* crtM*:*lpdA* double mutant also showed a decrease in its ratio in bacteria recovered from the infected liver and spleen compared to the ratio in the mixture used to infect the mice (Figure 5(c)). Survival of the* crtM*:*lpdA* appeared to be lower even compared to the* lpdA* mutants, at least in liver tissues (Figures 5(b) and 5(c)).

## 4. Discussion

Prevalence of the bacterium* S. aureus* in the environment facilitates colonization on human skin, nares, and other exposed tissues. Colonized individuals are at a greater risk of progressing to clinical infections [24–26]. Pathogenic bacteria, such as* S. aureus*, continuously face conditions that are hostile to their existence in the environment. Under conditions of low temperature, these pathogens are challenged to maintain the fluidity of their cellular membrane which to a large extent is regulated by the presence of branched-chain fatty acids (BCFAs). Production of BCFAs is dependent on a four-polypeptide-enzyme complex [27–29]. Inactivation of one of these polypeptide encodings genes,* lpdA*, resulted in significant decrease in membrane BCFAs, fluidity, growth at lower temperatures, and an increase in staphyloxanthin levels [11, 12]. Staphyloxanthin is a carotenoid pigment that likely functions as a virulence factor for* S. aureus* [13, 14] and at higher levels decreases membrane fluidity. Staphyloxanthin [16] also impacts the susceptibility of staphylococci to host antimicrobial peptides [16]. In this study, the* lpdA* mutant showed a more rigid membrane which is consistent with previous findings [11, 12]. The membrane fluidity of the staphyloxanthin deficient* S. aureus*, however, was almost comparable to the fluidity of the wild-type* S. aureus*. In addition,* S. aureus* cells deficient in both, BCFAs and staphyloxanthin, showed fluidity similar to* lpdA* mutants.

The role of staphyloxanthin in membrane fluidity of* S. aureus* has been discussed in multiple reports [15–18]. A slight increase in membrane fluidity was noted in staphyloxanthin deficient* crtM* mutant of* S. aureus *[16]. When this mutant was complemented with the entire* crtOPQMN* operon* in trans* resulting in a hyperpigmented strain of* S. aureus* with much higher levels of staphyloxanthin, these bacteria showed a much more rigid membrane [16]. Higher level of staphyloxanthin in* S. aureus* makes the membrane more rigid and this has been reconfirmed in several additional studies [15, 17]. When* S. aureus* strains JE2 and SH1000 were grown in Muller-Hinton broth (MHB) and Luria-Bertani (LB) broth, they produced higher levels of BCFAs compared to when grown in brain heart infusion broth, TSB, or fetal bovine serum [10]. Both strains, JE2 and SH1000, that produced higher BCFA in MHB, also produced higher levels of staphyloxanthin. However, the LB broth grown SH1000 and JE2 cells, with higher BCFA levels, showed no increase in their staphyloxanthin content [10]. Similarly, the MHB grown cells showed a higher polarization value in membrane fluidity experiments suggesting a rigid membrane but the LB broth grown cells showed no such decrease in fluidity [10]. The authors hypothesized that the increase in membrane fluidity was counterbalanced by increasing the level of staphyloxanthin in these bacteria [10]. However, this does not explain why elevated levels of staphyloxanthin was not seen in all of the cultures with increased BCFAs.* S. aureus* cells that lack a functional BKD enzyme, which is believed to be the main bacterial enzyme responsible for BCFA synthesis, showed higher levels of staphyloxanthin [12]. Overall, BCFA appears to be the main regulator of membrane fluidity in* S. aureus*.

In the analysis of the fatty acids in the cellular membrane, the* lpdA *mutant of* S. aureus* showed a decreased production of BCFAs, which is consistent with prior reports [11, 12]. On the other hand, the fatty acid composition of the* crtM* mutant was similar to the membrane fatty acids of the wild-type* S. aureus*. Fatty acid composition of the* crtM*:*lpdA *double mutant was deficient in BCFAs and similar to the fatty acid composition of the* lpdA* mutants. The findings suggest that there is no correlation between staphyloxanthin and BCFA levels in* S. aureus *which is consistent with prior reports [16, 17].

Survival of* crtM* and* lpdA* mutants was also investigated under* in vivo* conditions in an intraperitoneal mouse infection model. We found that the* lpdA* mutants and the* crtM*:*lpdA *double mutants had significantly reduced survival and were cleared from infected mice at a faster rate compared to the wild-type* S. aureus*. Mutation in* lpdA *gene has previously been shown to have a decreased survival in mice [11]. Although, the* lpdA* mutants showed slower* in vitro* growth, it is unlikely to be the sole reason for the poor recovery of these mutant bacteria from the mouse tissues. Similar CFUs were injected in case of the* lpdA*,* crtM*, or* crtM*:*lpdA* double mutants in combination with the wild-type bacteria. In addition, the recovery of total tissue CFUs was comparable in case of the three mixtures that were injected in mice. These experiments were concluded within 24 h and during this time* in vivo*, the bacteria faced a hostile environment. While a lower growth rate of the* lpdA* mutant might be a contributing factor, it is more likely that the decrease in membrane BCFAs puts the cells at a disadvantage under* in vivo* conditions. There was no change in the survivability of the* crtM* mutants in our investigations. However, previous studies have shown that the lack of staphyloxanthin increases the vulnerability of* S. aureus* cells to neutrophil killing [13, 30]. In that investigation, lack of staphyloxanthin reduced the virulence potential of* S. aureus* in a mouse skin abscess model [13].

The potential of staphyloxanthin as a drug target has recently been investigated [19]. When Naftifine was used to inhibit staphyloxanthin production (by inhibiting the activity of the enzyme encoded by* crtN*) in mice infected with methicillin-resistant staphylococci, these bacteria showed significantly reduced bacterial counts in kidneys and hearts of treated mice compared to untreated mice [19]. In contrast, the liver of the mice showed no change in bacterial load in Naftifine treated mice vs nontreated mice [19]. The* crtM *mutant used in this study showed no significant reduction in survival under* in vivo* conditions. This seems in contrast to the previous findings [13], and it may be due the strain used in this study (which is a methicillin-sensitive strain vs methicillin-resistant strain), the type of infection model (intraperitoneal vs skin abscess model), and the infected tissues that were analyzed. Nonetheless, the* crtM*:*lpdA *mutant showed an even greater decrease in survival compared to the individual* crtM *or* lpdA *mutants.

## 5. Conclusions

In summary, a mutation in the* lpdA *gene decreases the growth rate, membrane fluidity, and virulence of* S. aureus*. Mutation in the* crtM *gene eliminates pigment production, but this lack of staphyloxanthin has little to no effect on growth rate, membrane fluidity, or virulence of* S. aureus *strain SH1000. A double mutation in* crtM* and* lpdA* genes showed a much greater growth defect* in vitro*. These double mutants also showed reduced* in vivo* survival suggesting that the simultaneous targeting of the BCFA and staphyloxanthin metabolic pathways in* S. aureus* may be an attractive strategy to control infections caused by this pathogen.

## Figures and Tables

**Table 1:** Bacterial strains used in this study.

Strains	**Characteristics**	**Reference**
SH1000	A methicillin-sensitive and SigB positive *S. aureus *	[20]

SH1000:*lpdA*	*lpdA *mutant of *S. aureus* strain SH1000 (Kan^R^)	[11]

SH1000:*crtM*	Mutation in *crtM* gene transduced from NE1444 to *S. aureus* SH1000 (Erm^R^)	This study

SH1000:*crtM*:*lpdA*	A *crtM*:*lpdA* double mutant of *S. aureus* SH1000 (Kan^R^, Erm^R^)	This study

SH1000:*lpdA*+pCU-BKD	*lpdA* mutant of SH1000 complemented with BKD gene cluster *in trans *(Kan^R^, Cam^R^)	[11]

SH1000:*crtM*+pCU-*crtOPQMN*	*crtM* mutant of SH1000 complemented with *crt* gene cluster *in trans *(Erm^R^, Cam^R^)	This study

SH1000:*crtM*:*lpdA*+pCU-BKD	A *crtM*:*lpdA* double mutant of SH1000 complemented with BKD gene cluster (Kan^R^, Erm^R^, Cam^R^)	This study

SH1000:*crtM*:*lpdA*+pCU-*crtOPQMN*	A *crtM*:*lpdA* double mutant of SH1000 complemented with *crt* gene cluster (Kan^R^, Erm^R^, Cam^R^)	This study

**Table 2:** Generation time of different *S. aureus* strains.

Strain	Generation time (min)
SH1000	32.09
SH1000:*lpdA*	34.49
SH1000:*crtM*	32.68
SH1000:*crtM*:*lpdA*	39.51

**Table 3:** Major membrane fatty acids of *S. aureus* strains.

	**Wild-type SH1000**	***crtM *mutant**	***lpdA *mutant**	***crtM:lpdA***
**C10:0**	0.72 ± 0.20	0.66 ± 0.13	0.64 ± 0.40	0.67 ± 0.39

**C12:0**	0.60 ± 0.10	0.60 ± 0.09	0.56 ± 0.05	0.55 ± 0.04

**C13:0 iso**	0.25 ± 0.20	0.23 ± 0.02	0.42 ± 0.17	0.37 ± 0.17

**Iso C14:0**	2.60 ± 0.40	2.68 ± 0.20	8.13 ± 0.44	10.5 ± 0.33

**C14:0**	1.55 ± 0.40	1.61 ± 0.21	9.67 ± 1.92	7.62 ± 2.30

**Iso C15:0**	13.5 ± 0.90	13.59 ± 0.61	3.27 ± 0.05	3.81 ± 0.21

**Anteiso C15:0**	35.4 ± 2.60	35.5 ± 1.70	17.1 ± 5.93	19.4 ± 5.70

**Iso C16:0**	3.65 ± 0.30	3.64 ± 0.10	3.28 ± 0.76	4.55 ± 1.30

**C16:0**	6.20 ± 0.30	6.66 ± 0.22	16.2 ± 2.07	14.9 ± 2.34

**Iso C17:0**	4.14 ± 0.30	4.00 ± 0.13	0.41 ± 0.06	0.54 ± 0.14

**Anteiso C17:0**	7.86 ± 0.90	7.28 ± 0.57	1.65 ± 0.86	2.02 ± 1.20

**C17:0**	0.42 ± 0.03	0.36 ± 0.05	0.66 ± 0.10	0.27 ± 0.08

**Iso C18:0**	0.89 ± 0.10	0.85 ± 0.02	0.55 ± 0.19	0.66 ± 0.25

**C18:0**	12.0 ± 0.70	12.6 ± 0.59	20.9 ± 1.58	18.9 ± 2.23

**Anteiso C19:0**	1.11 ± 0.04	0.96 ± 0.05	0.35 ± 0.04	0.28 ± 0.13

**C19:0**	0.58 ± 0.02	0.45 ± 0.02	0.75 ± 0.11	1.21 ± 0.19

**C20:0**	5.44 ± 0.60	5.68 ± 0.89	10.7 ± 1.31	6.47 ± 0.92

**Anteiso fatty acids**	44.4	43.7	19.0	21.7

**Iso fatty acids**	26.3	26.2	16.1	20.4

**BCFA**	70.7	69.9	35.1	42.1

## Data Availability

The data used to support the findings of this study are included within the article.
